# Supplementary figures and images for: Camptothecin Effectively Regulates Germline Differentiation through Bam–Cyclin A Axis in Drosophila melanogaster
Source: Int J Mol Sci. 2023 Jan 13;24(2):1617. doi: 10.3390/ijms24021617 (PMC9864452; doi:10.3390/ijms24021617)

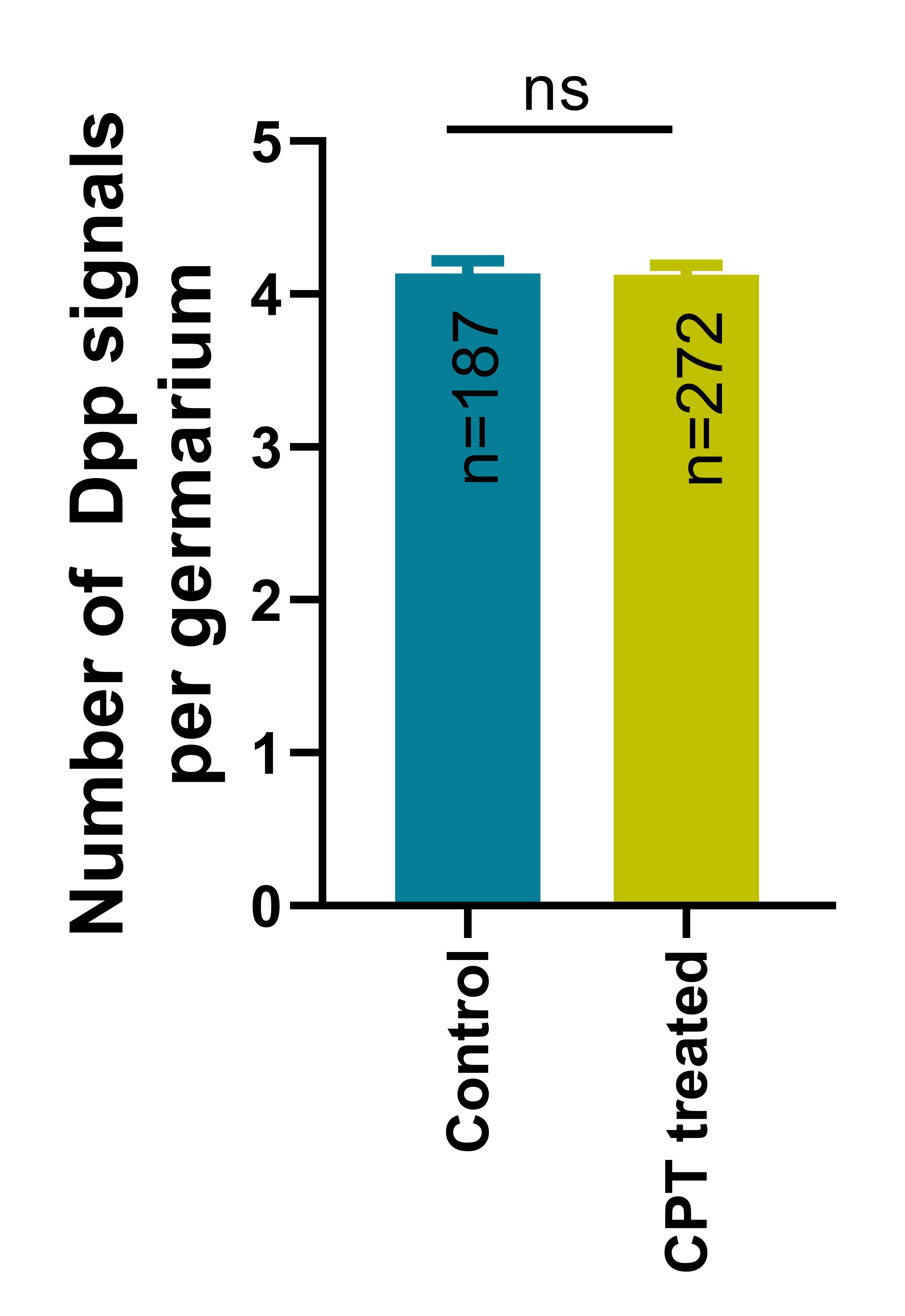

Supplement: Supplementary file 1 [file ijms-24-01617-s001.zip › ijms-2100871-Figure S1.jpg]
